# Resynchronization Therapy During Sustained Ventricular Tachycardia

**DOI:** 10.19102/icrm.2017.080103

**Published:** 2017-01-15

**Authors:** Sergio F. Cossú

**Affiliations:** ^1^The Arrhythmia Center at the Charlotte Heart and Vascular Institute, Port Charlotte, FL

**Keywords:** Biventricular implantable cardioverter-defibrillator, ventricular sense response, ventricular tachycardia, resynchronization therapy

## Abstract

A 62-year-old gentleman with a history of an ischemic cardiomyopathy and previous implantation of a biventricular cardioverter-defibrillator presented with complaints of palpitations and a wide complex ventricular paced rhythm at 120 bpm. This was originally thought to be ventricular tracking of an atrial tachycardia at the upper tracking rate, as the patient remained hemodynamically stable for three consecutive days in this rhythm. On the third day, the patient’s implantable cardioverter-defibrillator (ICD) was interrogated and it was found that he was indeed in a sustained ventricular tachycardia with biventricular pacing being delivered as a function of the ventricular sense response feature. When this feature was turned off, the patient immediately deteriorated hemodynamically and required a commanded shock through the ICD to terminate the tachycardia. This is an extremely rare presentation of this pacing feature found in biventricular ICDs, which in this case provided significant hemodynamic benefit during a malignant arrhythmia.

## Case presentation

**Figure 1:**
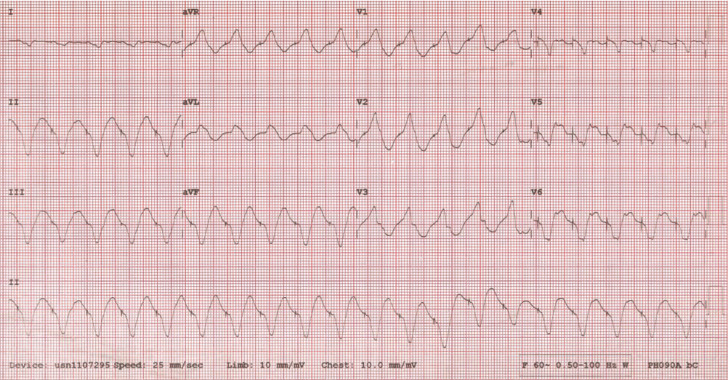
A 12-lead electrocardiogram that demonstrates a wide complex rhythm with pacemaker spikes preceding QRS complexes. The ventricular rate is approximately 440 ms (125 bpm).

A 62-year-old male patient with a history of an ischemic cardiomyopathy and a previously implanted biventricular cardioverter-defibrillator (Concerto II CRT-D, Medtronic Inc., St. Paul, MN) was admitted with complaints of palpitations and noted to have a blood pressure of 95/68 mmHg and a heart rate of 120 bpm. The patient also has a history of atrial fibrillation and had undergone catheter ablation of the atrioventricular (AV) node in the past at another institution. Since the time of his ICD implant, he has received several appropriate therapies for ventricular tachycardia and has been managed with amiodarone. An initial electrocardiogram (ECG) demonstrated a wide complex rhythm at a rate of 120 bpm with evidence of pacer spikes preceding the QRS complexes **(Figure 1)**.

The patient was initially believed to have an atrial tachyarrhythmia with ventricular tracking at the upper rate limit; he received further amiodarone and β-blocker therapy without any significant change in the heart rate. He remained otherwise hemodynamically stable in this rhythm without any complaints except for palpitations for 3 days following provision of the new regimen.

**Figure 2:**
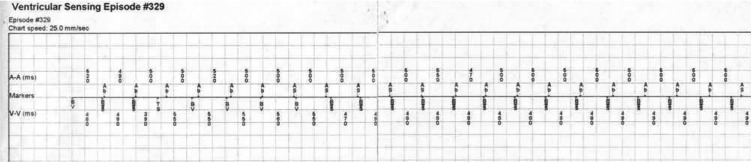
Intracardiac markers from the patient with the presenting arrhythmia’s implantable cardioverter-defibrillator demonstrate an ongoing ventricular sensed rhythm at a cycle length of 490–560 ms with atrioventricular dissociation and biventricular pacing on sensed beats. An atrial tachycardia at a cycle length of 500 msec is also evident. Markers are suggestive of a ventricular sense response algorithm. Ab = atrial blanking; as = atrial sensing; TS = ventricular tachycardia sense; BVS = biventricular pacing on a ventricular sensed beat.

**Figure 3:**
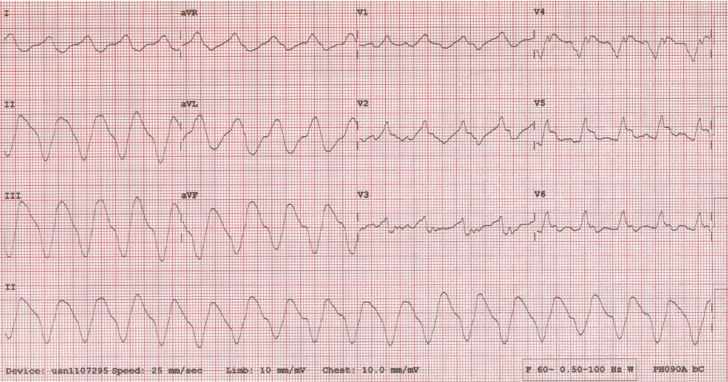
A 12-lead electrocardiogram with inhibition of pacing that demonstrates an underlying ventricular tachycardia at a cycle length of 540 ms (112 bpm) of a right bundle branch, left superior axis morphology.

Subsequent examination of the patient’s implantable cardioverter-defibrillator (ICD) demonstrated an ongoing ventricular sensed rhythm at a cycle length of 490–560 ms with AV isorhythmic dissociation and biventricular pacing on the ventricular sensed beats **(Figure 2)**. Pacing was then inhibited, which resulted in the ECG shown in **Figure 3** that demonstrates a bizarrely appearing wide complex tachycardia of right bundle branch morphology, left superior axis at a cycle length of 540 ms. Following discovery of this observation, the patient deteriorated hemodynamically, becoming hypotensive (74/55 mmHg). Attempts at anti-tachycardia pacing failed to terminate the tachycardia and he was subsequently rescued using a commanded shock through the ICD.

## Discussion

Modern technological advances in device therapy have also been fraught with the occurrence of complex pacemaker-mediated arrhythmias, which have led to a significant amount of confusion for the clinician.^1,2^ Various pacing algorithms intended to improve patient outcome can at times appear to be a device malfunction instead. The current case presents such a diagnostic dilemma, as it is clearly not a malfunction of the device. The patient was initially believed to have an atrial tachyarrhythmia with ventricular pacing at the upper tracking rate because of lack of appropriate mode switching. The other possibility would have been a pacemaker-mediated tachycardia, although most current ICDs have built-in safeguard features that are programmable to prevent and treat such arrhythmias. Knowledge of the patient’s previous history of AV nodal ablation would have made this less likely and knowledge of the patient’s programmed parameters also would have clearly assisted in the making of the appropriate diagnosis and the ruling out of any potential device-mediated arrhythmias. As such, the present case is unique in that it represents a ventricular tachyarrhythmia that is masked as a paced rhythm.

The ventricular sense response (VSR) algorithm attempts to maintain cardiac resynchronization therapy in the presence of rapidly conducted atrial fibrillation via the initiation of a biventricular paced event 1.25 ms following a sensed ventricular beat.^3,4^ If the device is programmed in a dual-chamber mode, as was the case with this particular patient with a left ventricular (LV) to RV offset, then a ventricular sensed event following an atrial sensed event would trigger the device to pace the LV 1.25 ms after the ventricular sensed complex, followed by the RV 2.5 ms later as demonstrated in **Figure 2**. Interestingly, the patient does appear to have a sinus/atrial tachycardia at a rate of 500 ms as shown in **Figure 2**, although there is clear-cut evidence of isorhythmic dissociation demonstrated consistent with a dual tachycardia.

Furthermore, analysis of the patient’s clinical arrhythmia upon inhibition of the device makes tracking of an atrial arrhythmia less likely. The biventricular paced rhythm that is delivered is at the programmed offset, which in this case was LV to RV. Newer devices allow for the programmability of LV only, RV only, or biventricular pacing during ventricular sense response. In this instance, the patient’s presenting rhythm was slower than the VT detection rate of 150 bpm and slower than the VSR maximum tracking rate of 130 bpm. The patient’s clinical VT, whose cycle length had significantly slowed as a result of amiodarone therapy, was sensed by the ICD as a ventricular sensed event rather than ventricular tachycardia, and therefore delivered biventricular pacing on top of it. As a result of this slower rate, the VSR algorithm actually entrained the tachycardia and allowed it to persist at this rate for three days. Indeed, it can be seen in **Figure 1** that the rate of the biventricular paced rhythm is clearly faster than the rate of the patient’s clinical ventricular tachycardia in **Figure 3**, with some morphological changes in the QRS, most notably in Leads I, V5 and V6. This observation suggests that there is some form of entrainment of the tachycardia with fusion because of pacing from a remote region of the infarct zone. This is also made evident by the narrower QRS morphology during pacing.

Analysis of the ECG during the patient’s ventricular tachycardia revealed the location of the ventricular tachycardia most likely originated from the inferior basal wall and was possibly epicardial in origin, because of the slurred and wide QRS morphology and a maximum deflection index of approximately 0.67 **(Figure 3)**. The patient’s LV lead was not implanted by the author; however, the patient’s chest X-ray did demonstrate the fact that a posterolateral branch of the coronary sinus had possibly been utilized for placement of the LV lead. Given the fact that we had QRS fusion of the paced complexes compared with the patient’s clinical ventricular tachycardia because of both RV and LV pacing, one cannot make any assumptions about the location of the coronary sinus lead in relation to the infarct zone.

Of interest is the fact that this proved to be of hemodynamic benefit to the patient, who remained stable in this rhythm during the entire time. Research has shown the clinical beneficial effects of VSR pacing in patients with atrial fibrillation or frequent ventricular ectopy via the maximization of biventricular pacing and thus improvement of resynchronization and heart failure symptoms.^3,4^ The VSR in this case provided improved hemodynamic support through resynchronization therapy by delivering a biventricular paced rhythm during a sustained ventricular tachycardia.

## Conclusion

In an ongoing effort to improve the efficacy of their productsand allow for more customizable therapy for better patient management, cardiac rhythm management manufacturers continue to develop complex algorithms that impact the appearance of a patient’s rhythm. As the number of these features increases, it becomes ever more difficult to assess the functionality of the devices as well as the rhythm status of the patient using only the appearance of surface electrograms. Clinicians must thus be familiar with all algorithms in cardiac rhythm management devices and, when they activate, the hierarchy between these algorithms and what effects they may have on the ECG and the patient.
